# Assessment of Collagen-Based Nanostructured Biomimetic Systems with a Co-Culture of Human Bone-Derived Cells

**DOI:** 10.3390/cells11010026

**Published:** 2021-12-23

**Authors:** Giorgia Borciani, Giorgia Montalbano, Priscila Melo, Nicola Baldini, Gabriela Ciapetti, Chiara Vitale-Brovarone

**Affiliations:** 1 Biomedical Science and Technologies Laboratory, IRCCS Istituto Ortopedico Rizzoli, 40136 Bologna, Italy; giorgia.borciani@ior.it (G.B.); nicola.baldini@ior.it (N.B.); gabriela.ciapetti@ior.it (G.C.); 2 Department of Applied Science and Technology, Politecnico di Torino, Corso Duca degli Abruzzi 24, 10129 Torino, Italy; giorgia.montalbano@polito.it (G.M.); priscila.soares@polito.it (P.M.); 3 Department of Biomedical and Neuromotor Sciences, University of Bologna, 40136 Bologna, Italy; 4 Laboratory for Nanobiotechnology, IRCCS Istituto Ortopedico Rizzoli, 40136 Bologna, Italy

**Keywords:** cell co-culture, osteoblasts, osteoclast precursors, biomimetic composite, bone tissue engineering, mesoporous bioactive glasses, hydroxyapatite, type I collagen

## Abstract

Osteoporosis is a worldwide disease resulting in the increase of bone fragility and enhanced fracture risk in adults. In the context of osteoporotic fractures, bone tissue engineering (BTE), i.e., the use of bone substitutes combining biomaterials, cells, and other factors, is considered a potential alternative to conventional treatments. Innovative scaffolds need to be tested in in vitro systems where the simultaneous presence of osteoblasts (OBs) and osteoclasts (OCs), the two main players of bone remodeling, is required to mimic their crosstalk and molecular cooperation. To this aim, two composite materials were developed, based on type I collagen, and containing either strontium-enriched mesoporous bioactive glasses or rod-like hydroxyapatite nanoparticles. The developed nanostructured systems underwent genipin chemical crosslinking and were then tested with an indirect co-culture of human trabecular bone-derived OBs and buffy coat-derived OC precursors, for 2–3 weeks. The favorable structural and biological properties of the materials proved to successfully support the viability, adhesion, and differentiation of cells, encouraging a further investigation of the developed bioactive systems as biomaterial inks for the 3D printing of more complex scaffolds for BTE.

## 1. Introduction

Osteoporosis (OP) is classified as one of the world’s most common chronic metabolic bone diseases [1], characterized by a reduction of bone mass, low bone mineral density (BMD), and impaired bone microarchitecture/mineralization. These factors lead to increased bone fragility, which results in low-trauma fractures [2,3,4], especially in the elderly population. These fractures are age-related [5,6], therefore their numbers are expected to escalate due to the increasing aging population, making OP a major public health burden. Current treatments for reducing the risk of osteoporotic fractures can be summarized in antiresorptive and anabolic drugs. Antiresorptive drugs such as bisphosphonates (BPs) are amongst the most used compounds in the prevention and treatment of osteoporotic fractures [7]. Despite their success, long-term treatment with BPs can cause osteonecrosis of the jaw and atypical femur fractures [8,9,10]. This negative impact is related to the diminished capacity of fracture healing in osteoporotic patients, and the use of fracture fixation devices such as screws or fixation plates. In this scenario, a potential alternative to the conventional treatments is bone tissue engineering (BTE), which aims to induce the regeneration of new functional bone tissue through a combination of biomaterials, cells, and bioactive factors [11,12].

BTE focuses on mimicking the biological milieu to promote an effective regeneration of bone tissue, involving the coordinated action of osteoblasts (OBs) and osteoclasts (OCs). Accordingly, several works have underlined the key role of OB/OC crosstalk and coupling during the process of bone formation and resorption [13]. OBs and OCs strictly cooperate in a coordinated spatio-temporal manner to complete bone remodeling and bone regeneration processes even if the OCs and OBs are not in direct contact, as proven by the physical connection existing between resorption and new bone formation through the reversal phase [14]. Frequently, new materials for BTE applications are evaluated by using only one cell type, missing the advantages of co-culture vs. monoculture models. Co-culture models reproduce the in vivo environment closer than the monoculture models as they grant cells additional functions due to intercellular signal transmission through paracrine activities among the different cell types [13]. The significance of setting up a co-culture of OBs and OCs, for both material and scaffold testing, is to provide an ex vivo model for a detailed examination of the cellular crosstalk and their signaling pathways during the remodeling process.

In this study, the authors tried to foster bone engineering techniques through a better understanding of the coupling mechanism between bone cells when testing new material. In order to reproduce the natural bone microenvironment, composite systems, based on type I collagen, were prepared, as collagen can sustain cells and act as a template to promote bone regeneration [15]. To modify the biomechanical properties of the final biomaterial, and increase its bioactive characteristics [16], inorganic phases capable of inducing cells mineralization, e.g., hydroxyapatite and bioactive glasses (BGs), were added [17,18]. In previous studies, the authors presented composite systems containing either mesoporous BGs incorporating 4% mol. of strontium (Sr^2+^) (MBG_Sr4%) or rod-like nanohydroxyapatite (nano-HA), both biocompatible materials able to induce cell differentiation and mineralization [16]. Since natural collagen has a high degradation rate in vivo and lacks biomechanical stiffness, the structures often undergo crosslinking, either physical, chemical, or both [19]. The authors optimized a chemical crosslinking strategy for collagen-based hydrogels using a biocompatible natural agent, genipin, a plant extract from the gardenia fruit [20], a method proved to increase the material’s stiffness without cytotoxic effects. Even though these results and the ones presented in the literature are promising, they are related to the use of only one cell type when testing the biomaterials, therefore disregarding the interaction with other cells in the surroundings. In the case of OP, this could be unsatisfactory as the signs of OP are caused by an imbalance in bone resorption and formation [21], and a good availability of local osteoprogenitors at the bone defect sites is the key to ensuring a positive outcome [22]. Moreover, the role of osteoblast-osteoclast communication and coupling during bone remodeling is essential to promote optimal regeneration [23]. In this light, this study is focused on the assay of biomimetic and bioactive composite materials that promote bone regeneration using a bone cell co-culture. Using previously studied materials, the authors designed a system where the material would interact with bone cells simultaneously. To this aim, the material has been tested in a co-culture of human OBs and OCs: OBs were isolated from trabecular bone and seeded in direct contact with the bulk material, while precursors of OCs were placed into transwell devices introduced into the culture wells. This indirect co-culture system was set up in order to detect the OB/OC interaction, as well as the OC differentiation induced by paracrine signaling [13].

The main features of the developed composite systems were initially investigated by performing morphological, rheological, and degradation evaluations. The two composite materials were subsequently tested in presence of the co-culture system to assess their potential influence over OB and OC activities while demonstrating that the developed co-culture model can represent a promising screening tool to test novel smart materials for BTE. In detail, cell viability was assessed by means of Alamar Blue and Live/Dead assays, while Scanning Electron Microscopy (SEM) was used for the morphological assessment. The alkaline phosphatase (ALP) activity of OBs was monitored, while OC formation was checked using specific OC markers, including Tartrate Resistant Acid Phosphatase (TRAP) staining, Phalloidin-fluorescein isothiocyanate (FITC) label for actin filaments, and Hoechst stain for multinuclearity. The results suggest that the developed composite systems support bone cells and possess suitable biomechanical properties, deserving further refinement to act as bone scaffolds prepared by 3D printing techniques.

## 2. Materials and Methods

### 2.1. Preparation of the Collagen-Based Composite Systems (Coll/MBG_ Sr4% and Coll/Nano-HA)

Collagen-based formulations containing MBG_Sr4% produced via the sol-gel route (Coll/MBG_Sr4%) or rod-like hydroxyapatite nanoparticles (Coll/nano-HA), were synthesized following protocols previously designed by the authors and reported in detail in the previously published works [20,24,25]. Briefly, the Coll/MBG_Sr4% suspension was obtained by adding MBG_Sr4% nanoparticles to 0.5 M acetic acid and then mixing them with a 1.5% wt collagen solution (bovine collagen from Achilles tendon; Blafar Ltd., Dublin, Ireland). The pH was then neutralized with 1 M NaOH. Similarly, for the Coll/nano-HA system, nano-HA particles were suspended in 1 M NaOH containing an anionic dispersing agent (Darvan 821-A), then the suspension was added to the 1.5% wt collagen solution. The relative amount of collagen powders and inorganic phases were calculated based on the ratio and volume percentages of the organic and inorganic constituents of bone, where collagen and hydroxyapatite count for 53% vol. and 47% vol., respectively [26].

To obtain solid bulk samples of Coll/MBG_Sr4% and Coll/nano-HA, 400 μL of the specific suspension were pipetted in a silicon mold, then subsequently incubated at 37 °C for 3 h in order to allow the sol-gel transition of the system arising from the physical crosslinking of collagen molecules [20]. The solid samples were further chemically crosslinked by incubation in a solution of 0.5% wt genipin in 70% ethanol (GEN/EtOH) at 37 °C for 24 h.

### 2.2. Characterization of the Collagen-Based Composite Systems

#### 2.2.1. Morphological and Physico-Chemical Assessment by Means of Field Emission Scanning Electron Microscopy (FESEM)

Before sample examination by Field-Emission Scanning Electron Microscopy (FESEM), GEN/EtOH crosslinked Coll/MBG_Sr4% and Coll/nano-HA bulk samples were frozen at −20 °C and lyophilized for 24 h using a Lyovapor L-200 freeze-dryer (Büchi, Flawil, Switzerland) under vacuum (<0.1 mbar). For the morphological assessment, the lyophilized samples were coated with a 7 nm thin platinum layer and analyzed using a ZEISS MERLIN FESEM instrument.

#### 2.2.2. Rheological Properties

The viscoelastic properties of the two composite systems were studied by means of rheological tests, monitoring the variation of the storage (G′) and loss (G″) moduli at different stress and temperature conditions. The composites were tested by using a DHR-2 rotational Rheometer (TA Instruments, Waters, New Castle, PA, USA) equipped with a parallel plate geometry with a diameter of 20 mm and a Peltier plate to constantly control the system’s temperature.

The material strength was assessed by dynamic amplitude sweep tests by setting a range of 0.01–1% strain at a constant frequency of 1 Hz, registering G′ and G″ values.

Oscillatory temperature ramps ranging between 25 °C and 80 °C were performed under 1% strain and 1 Hz using a ramp rate of 5 °C/min to detect the denaturation temperature of the material associated with the sharp decrease of the overall viscoelastic moduli (G′, G″).

#### 2.2.3. In Vitro Enzymatic and Hydrolytic Degradation

The stability of the crosslinked samples was tested by using both hydrolytic and enzymatic degradation tests. The percentage of weight loss was measured at different time points after incubation in Dulbecco phosphate-buffered saline (D-PBS) and collagenase solutions, comparing the two different systems. Collagenase from Clostridium histolyticum (type I, Sigma-Aldrich, Milan, Italy) was used for the enzymatic degradation study of the different systems, immersing each sample in 1 mL of Dulbecco’s Modified Eagle’s Medium (DMEM, Sigma-Aldrich, Milan, Italy) containing 1 mg of collagenase (2.1 units), and subsequently incubating them at 37 °C under mild agitation (50 rpm) At predefined time points (12, 24, and 48 h) samples were collected and thoroughly washed in distilled water. D-PBS was selected as the medium for hydrolytic degradation tests, where samples were immersed, in 3 mL of solution, then incubated at 37 °C under mild agitation (50 rpm) At predefined time points (24 h, 72 h, 7 days, and 14 days) the samples from both degradation routes were collected, frozen, lyophilized, and accurately weighted to record the final weight. The total weight loss was assessed by using the following formula:

W (%) = [(W_0_ − W_d_)/W_0_] × 100 (1)

where W_0_ represents the initial mass of the sample, W_d_ is the mass after degradation, and W is the resultant loss. Three samples were tested for each time point, and results were reported as mean ± standard deviation.

### 2.3. In Vitro Biological Assessment: Indirect Co-Culture System

#### 2.3.1. Osteoblasts Isolation and Culture

OBs were isolated from trabecular bone samples of patients undergoing surgery for fracture repair or other orthopedic procedures, following the patient’s written informed consent approved by the Institutional Ethical Committee. The trabecular bone sample was dissected into tiny fragments, washed twice with PBS, and incubated in 25 cm^2^ culture flasks with Ca^2+^-free DMEM-Low Glucose (DMEM-LG, Gibco, Thermo Fisher Scientific, Milan, Italy) (to inhibit fibroblast growth), supplemented with 10% fetal bovine serum (FBS, Sigma, Milan, Italy), 100 U/mL penicillin and 100 μg/mL streptomycin (Euroclone, Milan, Italy), and 2 mm l-glutamine (Euroclone, Milan, Italy). Cells were then incubated at 37 °C, in a 5% CO_2_ humidified atmosphere and the medium changed every 3 days.

When OBs reached confluence, they were detached by means of trypsin-EDTA (Euroclone, Milan, Italy) digestion. Second- to six-passage cells were used for the experiments.

#### 2.3.2. Peripheral Blood Mononuclear Cell Isolation and Culture

Peripheral blood mononuclear cells (PBMCs) were isolated from the buffy coat of healthy donors by means of the Ficoll-Paque (GE Healthcare, Milan, Italy) density-gradient method, following the patient’s written informed consent approved by the Institutional Ethical Committee. After a 1:1 dilution of blood with PBS, 4 mL of diluted blood was layered on top of 3 mL Ficoll-Paque in 15 mL-conical tubes. Following several gradient centrifugations, the PBMCs were resuspended in DMEM–High Glucose (Sigma-Aldrich, Milan, Italy) supplemented with 10% FBS (Euroclone, Milan, Italy) (complete DMEM-HG) and seeded at a density of 3 × 10^6^/cm^2^ on the polyethylene terephthalate (PET) transwell (with 0.4 μm pore size) (SARSTEDT, Nümbrecht, Germany).

#### 2.3.3. Indirect Co-Culture Set Up with Coll/MG_Sr4% and Coll/Nano-HA Systems

**Figure 1:**
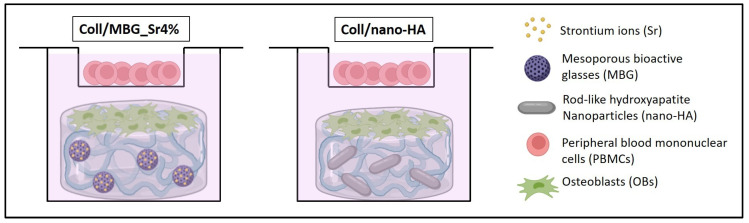
The set-up of the indirect co-culture within the materials. Osteoblasts (OBs) are seeded on the surface of the material and peripheral blood mononuclear cells (PBMCs) are co-cultured in the transwell device.

A thin biomaterial layer covering the entire bottom of the well was obtained by placing 400 µL of the hybrid formulations per well, into a 24 multi-well plate. Once prepared, the multi-well plate was incubated for 3 h at 37 °C, with 95% humidity to enable the biomaterial physical crosslinking. Then, the chemical crosslinking was accomplished by adding 2 mL of 0.5% GEN/EtOH solution to each biomaterial-seeded well at 37 °C, with 95% humidity for 24 h. Samples were washed in PBS twice using orbital shaking at 100 rpm for 30 min. The pre-wetting was obtained by adding 1 mL of 1:1 DMEM-HG and DMEM-LG (basal medium, BM) to each sample, then incubating them at 37 °C, with 95% humidity for 2 h. After resuspension in 500 µL of DMEM-LG, 5 × 10^4^ OBs were seeded on each material sample. At the same time, 1 × 10^6^ freshly isolated PBMCs were resuspended in 250 µL of DMEM-HG and seeded into the transwell. After 4 h, to allow cell adhesion, the indirect co-culture system was set up by placing the transwell with PBMCs inside the OB-seeded well, with 2 mL of BM added and refreshed every 3 days. The schema of the final set-up of the co-culture within the materials is presented in Figure 1.

#### 2.3.4. Assessment of Cell Viability

Cell viability was evaluated with Alamar Blue, for the presence of metabolically active cells, and Live Dead assay, to distinguish viable cells from dead ones.

The one-step Alamar blue assay (Invitrogen, Carlsbad, CA, USA) was applied separately to OBs and PBMCs. According to the manufacturer’s instructions, the culture medium was removed, replaced with the Alamar Blue solution prepared as 10% *v*/*v* in fresh BM, then incubated at 37 °C, with 95% humidity for 4 h. The fluorescence was quantified in a microplate spectrophotometer (Infinite F200 PRO, TECAN, Mannedorf, Switzerland) at 535 nm excitation and 590 nm emission wavelengths. Data expressed as Relative Fluorescence Units (RFU), are reported as mean ± standard deviation of triplicates. The hybrid material without cells was analyzed and considered as background, to be subtracted from the experimental samples.

The Live Dead assay (LIVE/DEAD™ Cell Imaging Kit (488/570), Invitrogen, Thermo Fisher Scientific, Milan, Italy) was applied to the OB population. Following the culture medium removal, the samples were washed with PBS, the staining solution was added, and the samples were incubated in an orbital shaker for 20 min at 37 °C, protected from light. After rinsing with PBS, the cells were examined in an optical fluorescent microscope (Nikon, Eclipse 800) and representative images were acquired at different magnifications using the NIS Element image software BR4.00.00 (Nikon).

#### 2.3.5. Indirect Cytotoxicity Test

To detect any potential toxic effect of the material’s degradation by-products, the indirect co-culture (with OBs in the wells and PBMCs in the transwells) was added for 24 h with the conditioned medium (CM) obtained from the materials kept in BM for 24 h. The maintenance of the cell co-culture system in CM allows detecting if degradation by-products released from the materials in the medium are able to influence cell viability. In this assay, cells are not seeded on the materials to exclude the direct effect of the materials on cell viability.

To perform the assay, a multi-well plate containing only the materials was maintained in BM at 37 °C, with 95% humidity for 24 h. After collection, the CM was diluted 100:0, 50:50, 25:75, and 5:95 with fresh BM, then added to the indirect co-culture. After 24 h, the viability of the cells in the co-culture with the four CM dilutions was assessed using the Alamar blue assay (as in Section 2.3.4.).

#### 2.3.6. Cell Morphology with Scanning Electron Microscopy (SEM)

Cell-seeded samples for SEM analysis were fixed in 2% glutaraldehyde (MERCK, Darmstadt, Germany) in 0.1 M sodium cacodylate buffer (Sigma-Aldrich, St. Louis, MO, USA), followed by washing in PBS and post-fixation in 1% osmium tetroxide (Electron Microscopy Sciences, Hatfield, PA, USA) in 0.1 M sodium cacodylate buffer. Complete dehydration was achieved in graded alcohol series and hexamethyldisilane. Samples were sputter-coated with platinum (up to 7 nm thickness) and the images were acquired with a Desktop SEM Phenom XL (Phenom-World B.V., Eindhoven, The Netherlands) at an accelerating voltage of 15 kV.

#### 2.3.7. Alkaline Phosphatase (ALP) Activity

ALP activity was measured at 7 and 14 days of culture, both in the supernatant of the indirect co-culture and in the OB-cell lysate. After collection of the supernatant, the OBs seeded on the biomaterial surface were lysed with Triton X-100 (Sigma-Aldrich, Milan, Italy) 0.1% in Tris buffer (pH 7.5). By mixing p-nitrophenyl phosphate with supernatant and cell lysate samples, the ALP activity was detected and quantified at 405 nm by means of a microplate spectrophotometer (Infinite F200 PRO, TECAN, Mannedorf, Switzerland).

#### 2.3.8. Assessment of Osteoclast Precursor Maturation

To evaluate the differentiation of PBMCs towards OCs, Tartrate Resistant Acid Phosphatase (TRAP) staining (Sigma-Aldrich, Milan, Italy), Phalloidin-fluorescein isothiocyanate (FITC, Sigma-Aldrich, Milan, Italy) label for cytoskeletal actin evaluation, and Hoechst (Sigma-Aldrich, Milan, Italy) stain for multinuclearity were used. For TRAP staining, after fixation with 3% paraformaldehyde (Sigma-Aldrich, Milan, Italy) and 2% sucrose (Electron Microscopy Sciences, Hatfield, PA, USA) in PBS, the cells were treated with a HEPES-Triton solution for 5 min for cell membrane permeabilization. After rinsing with PBS, the cells were incubated with the TRAP staining solution for 1 h at 37 °C, protected from light. For actin evaluation, cells were fixed with 3.7% paraformaldehyde in PBS and the cell membrane permeabilized with 0.5% Triton X-100 in PBS for 10 min. Subsequently, cells were incubated with FITC-conjugated Phalloidin solution in PBS, for 45 min away from light. Finally, for nuclear staining, after fixation with 3.7% paraformaldehyde in PBS and permeabilization in 0.5% Triton X-100 in PBS for 10 min, the cells were rinsed with PBS and incubated with 1.25 µg/mL Hoechst 33,258 for 10 min, protected from light.

### 2.4. Statistical Analysis

Data are expressed as mean ± standard deviation of three replicates, with *p* ≤ 0.05 considered as statistically significant. Statistical comparisons in the biological assays, between the experimental groups and between the different time-endpoints, were made by the nonparametric Mann–Whitney test for unpaired data, using the StatView 5.01 for Windows software (SAS Institute Inc., Cary, NC, USA).

## 3. Results

### 3.1. Physico-Chemical and Structural Properties of the Collagen-Based Biomimetic Systems

**Figure 2:**
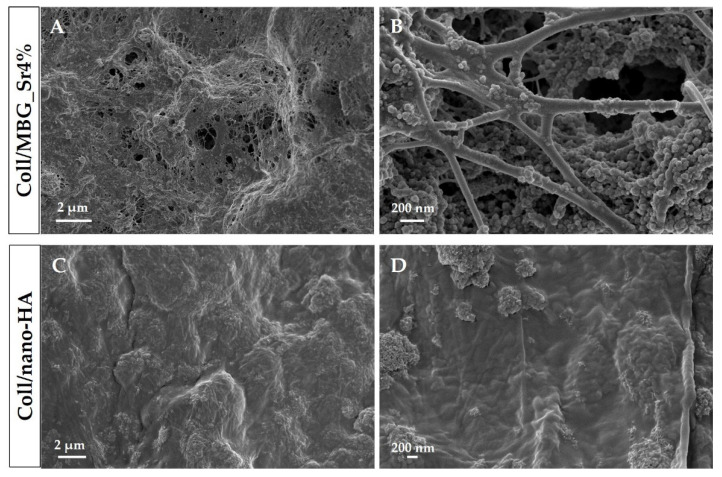
Cross-sectional FE-SEM images showing Coll/MBG_Sr4% and Coll/nano-HA samples at different magnifications.

Type I collagen is the main organic component of the bone extracellular matrix and is able to form nano- and micro-fibrillar structures due to the self-assembly of the molecules under physiological conditions of pH (7.4) and temperature (37 °C) [20]. Based on these observations and considering the composite nature of bone, type I collagen were separately combined with MBG_Sr4% and nano-HA, to obtain biomimetic and [20,25], the specific surface area of about 550 m^2^/g, and an average pore diameter of about 4 nm. The nano-HA was characterized by rods 40–60 nm long and 20 nm wide, presenting a sub-stoichiometric composition with a Ca/P ratio equal to 1.5 [24]. By mixing 1.5% wt type I collagen solutions with MBG_Sr4% or nano-HA, homogeneous systems of Coll/MBG_Sr4% and Coll/nano-HA were formed, respectively. FE-SEM images (Figure 2) confirmed the ability of the collagenous systems to reconstitute in fibrous solid matrices at physiological temperature and pH (37 °C, 7.4), where the formation of nano-sized fibrils due to the physical self-assembly of collagen was further supported by the genipin-induced chemical crosslinking.

Moreover, the morphological assessment showed the homogeneous distribution of both MBG_Sr4% and nano-HA embedded in the fibrillar collagenous matrix, without evidencing the presence of particle agglomerates greater than 1 µm in size.

**Figure 3:**
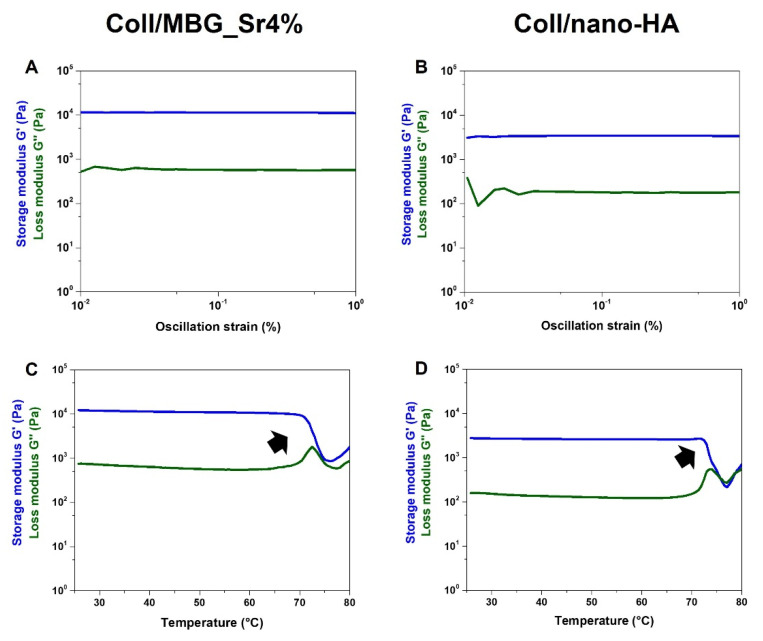
Viscoelastic properties of the composite systems: material strength (**A**,**B**) and denaturation temperature (**C**,**D**).

Besides the morphological features at the micro and nanoscale, rheological tests (Figure 3) were used to compare the strength and stability of the developed bioactive composites.

**Figure 4:**
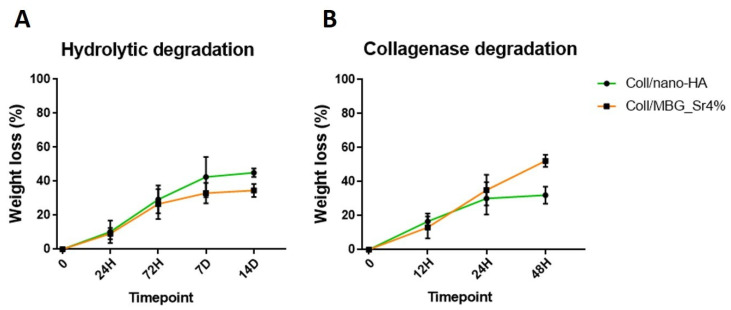
Hydrolytic (**A**) and enzymatic (**B**) degradation of Coll/MBG_Sr4% and Coll/nano-HA systems.

As represented in Figure 3A, an amplitude sweep test, performed at 37 °C on the Coll/MBG_Sr4% system, gave mean values of G′ and G″ of about 11,032 Pa and 599 Pa, respectively. In parallel, Coll/nano-HA (Figure 3B) samples presented a lower material stiffness, with G′ and G″ measuring about 3215 Pa and 229 Pa, respectively. The relevant gap between G′ and G″, visible in both graphs, is indicative of a predominant elastic behavior, different from what is normally observed for soft collagen hydrogels [12,27]. Moreover, the chemical crosslinking of collagen induced a significant increase in the material denaturation temperature, as clearly reported in Figure 3C,D. In detail, Coll/MBG_Sr4% and Coll/nano-HA subjected to a temperature ramp in the 25–80 °C range, underwent a sharp decrease of the overall complex modulus of the material between 70–73 °C, indicative of a structural collapse. Even if both composite systems presented high values of matrix strength, when compared to the MBG-containing system, the stiffness of Coll/nano-HA was found to be lower. As previously reported, carboxylate groups of collagen may interact with the calcium ions from hydroxyapatite, strongly reducing the interactions between genipin and collagen molecules, resulting in a lower degree of crosslinking [28]. To further investigate the stability of the developed composite systems, the hydrolytic and enzymatic degradation of Coll/MBG_Sr4% and Coll/nano-HA was monitored and compared (Figure 4).

The results shown in Figure 4 suggest a high resistance of both Coll/MBG_Sr4% and Coll/nano-HA to degradation in an aqueous saline medium as well as in an enzymatic solution, indicative of the effective formation of covalent bonds between collagen molecules upon genipin treatment [12]. The composite systems showed high stability in the aqueous environment (Figure 4A), registering less than 30% and 40% of weight loss after 14 days, for Coll/MBG_Sr4% and Coll/nano-HA, respectively. According to Figure 4B, after 48 h of incubation, Coll/MBG_Sr4% and Coll/nano-HA retained up to about 40% and 60% of the initial weight.

### 3.2. Biological Assessment of Indirect Co-Culture

#### 3.2.1. Cell Viability with Alamar Blue

**Figure 5:**
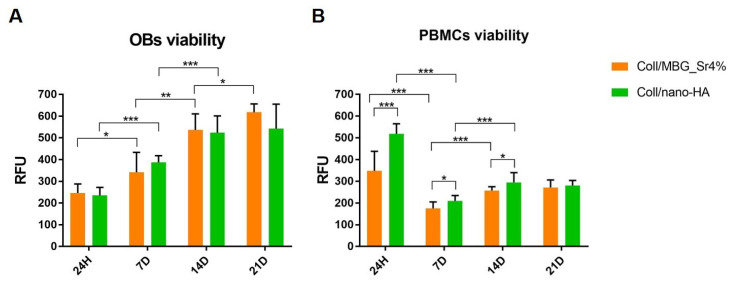
Histograms of the Alamar Blue assay showing OBs (**A**) and PBMCs (**B**) cell viability on the composite systems at the different time points (* *p*-value ≤ 0.05, ** *p* ≤ 0.01, *** *p* ≤ 0.001).

The results of the cell viability of OBs and PBMCs maintained in indirect co-culture with the composite materials are shown in Figure 5. The value of the biomaterial without cells was considered as background and subtracted from the RFU of the material-exposed cells.

The results of the Alamar Blue reduction confirmed the viability of OBs and PBMCs for both the composite systems at all the considered time points. Figure 5A clearly shows a statistically significant increase of OBs viability over time, with a similar trend for both materials. Indeed, no statistical differences between Coll/nano-HA and Coll/MBG_Sr4% were registered at the different time points.

In the case of PBMCs (Figure 5B), the high value of cell viability at 24 h, especially for Coll/nano-HA, decreased by day 7. However, cells recovered significantly by day 14, and the results were maintained up to day 21. Concerning PBMCs viability, statistically significant differences were observed at 7 days and 14 days between the two materials, with higher viability recorded for Coll/nano-HA.

#### 3.2.2. Cell Viability with Live Dead Assay

**Figure 6:**
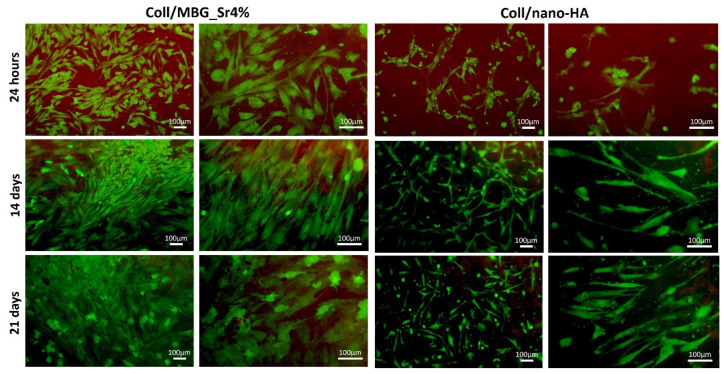
Representative images of OBs stained with the Live Dead assay, showing good cell viability at the different time points.

The results of the metabolic activity by the Alamar Blue assay were further confirmed by the Live Dead assay (Figure 6), which also allowed to appreciate the morphology of the OBs seeded on the composite materials (green: live cells; red: dead cells). Both surfaces showed adequate cell colonization, with most OBs viable from 24 h, and only a few spots of dead cells detected in the micrographs. On Coll/MBG_Sr4% the cells seem to be larger and flattened, with almost all the available surface of the material evenly covered after 21 days, whilst for Coll/nano-HA the spindle-shaped cells were fewer at this last time point.

#### 3.2.3. Indirect Cytotoxicity Test

**Figure 7:**
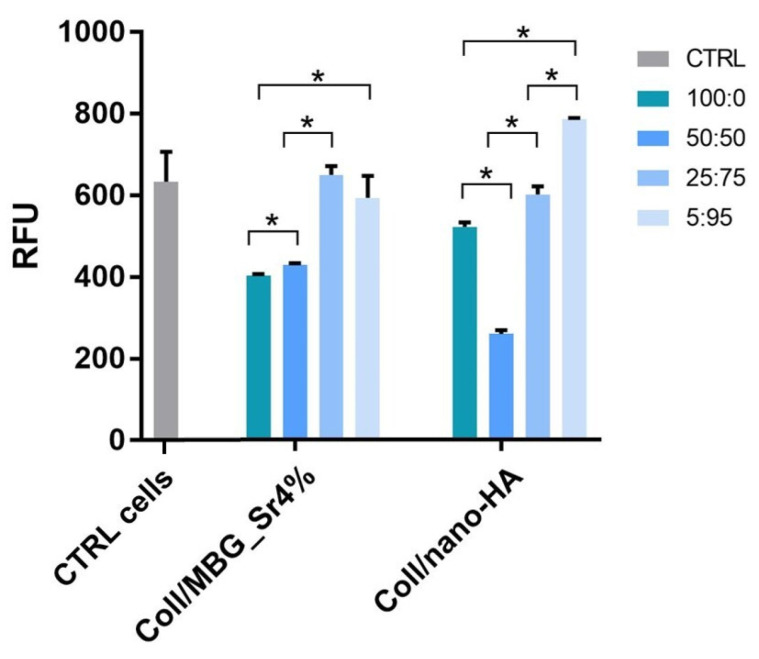
Cell viability by the Alamar Blue assay applied on the indirect co-culture maintained for 24 h in the conditioned medium (CM) diluted 100:0, 50:50, 25:75, and 5:95 with fresh culture medium. Only the *p* ≤ 0.05 was considered as statistically significant. Statistical analysis among CTRL cells and the other samples was performed as follows: CTRL cells and CM 100:0, 50:50; and 25:75 of Coll/MBG_Sr4% *p* = 0.0495; CTRL and CM 5:95 of Coll/MBG_Sr4% *p* = 0.517; CTRL and CM 100:0, 50:50, 25:75, and 5:95 of Coll/nano-HA *p* = 0.0495 (* *p*-value ≤ 0.05).

Figure 7 shows the cell viability by Alamar Blue assay of the indirect co-culture maintained for 24 h with the conditioned medium (CM) from the two composite materials, following CM dilution 100:0, 50:50, 25:75, and 5:95 with fresh basal culture medium. Data collected were compared to control cells (CTRL cells), that is the indirect co-culture maintained in a complete fresh culture medium for 24 h.

Cell viability decreased for concentrations above 50:50 dilution in both the composite materials but remained unaffected with higher dilutions, 25:75 and 5:95. For the material containing nano-HA, the viability registered for 5:95 dilution was slightly enhanced compared to the control.

#### 3.2.4. Cell Morphology with SEM

**Figure 8:**
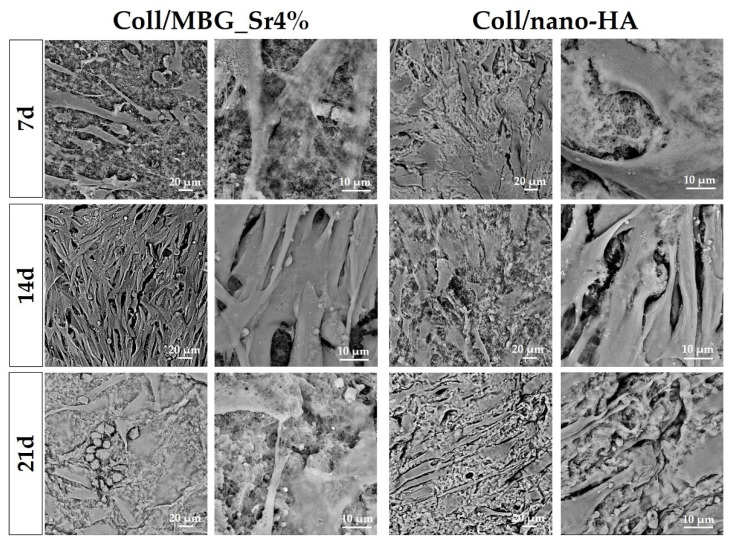
SEM micrographs of OB adhesion on Coll/MBG_Sr4% and Coll/nano-HA composite systems at the considered timepoints.

Adhesion and morphology of OBs on the two biomaterials were assessed by SEM (Figure 8). Elongated OBs were observed on Coll/MBG_Sr4% material surface proving their adhesion and presenting filopodia and membrane protrusions for cell-material attachment after 7 days. By day 14, an increased number of stretched OBs was observed, with several cell-cell contacts. After 21 days, round-shaped cells were dispersed among the spread cells, as well as mature OBs commonly represented by the cuboidal shape [29]. OBs on Coll/nano-HA show less stretching and branched processes at the early stage. However, spreading with more cell-cell contacts was observed at a later stage, confirming the good cell adhesion on this material.

**Figure 9:**
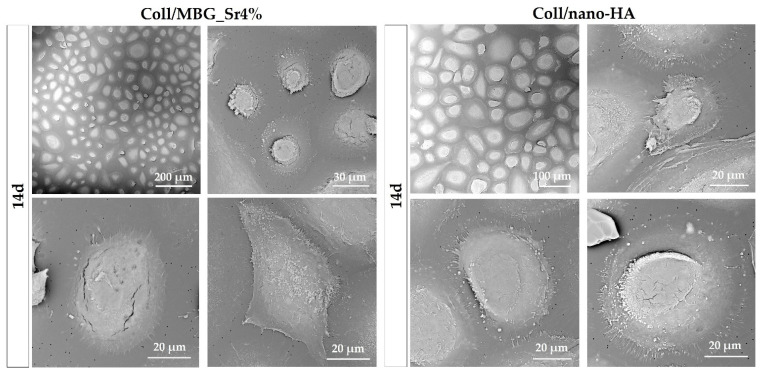
SEM micrographs of PBMC adhesion and differentiation in co-culture with Coll/MBG_Sr4% and Coll/nano-HA composite systems at the final time point.

Looking at the PBMC morphology (Figure 9), a higher frequency of large well-spread cells was observed for Coll/nano-HA in comparison to Coll/MBG_Sr4%. At higher magnifications, PBMCs on Coll/nano-HA showed a rounded morphology, with more evident ruffles in comparison to Coll/MBG_Sr4%, where PBMCs exhibit diverse shapes with minor cellular spreading and protrusions.

#### 3.2.5. Alkaline Phosphatase (ALP) Activity

**Figure 10:**
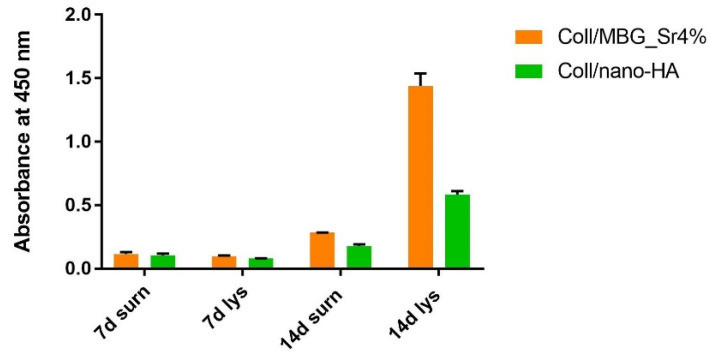
Diagram of the ALP activity at 7 and 14 days in the supernatant (surn) and cell lysate (lys) of OBs seeded on Coll/MBG_Sr4% and Coll/nano-HA in the indirect co-culture.

The ALP activity was measured in the culture supernatant (surn) and the cell lysate (lys) at 7 and 14 days (Figure 10).

ALP increased in the supernatants and cell lysates for both materials after 14 days, with the Coll/MBG_Sr4% sample showing the highest value for the cell lysate.

#### 3.2.6. Assessment of Osteoclast Precursor Maturation

**Figure 11:**
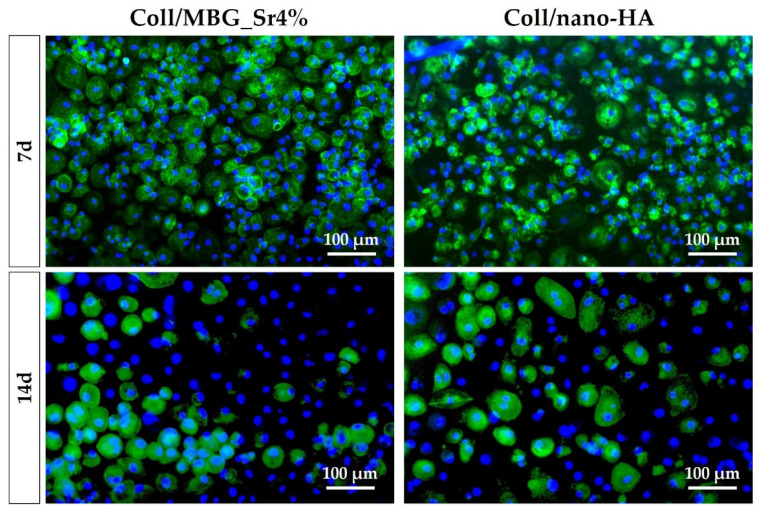
Fluorescence micrographs of PBMCs/pre-OCs on transwells co-cultured with OB-loaded materials at 7 and 14 days. Cytoskeletal actin and multinuclearity are stained with Phalloidin-FITC and Hoechst 33258, respectively.

**Figure 12:**
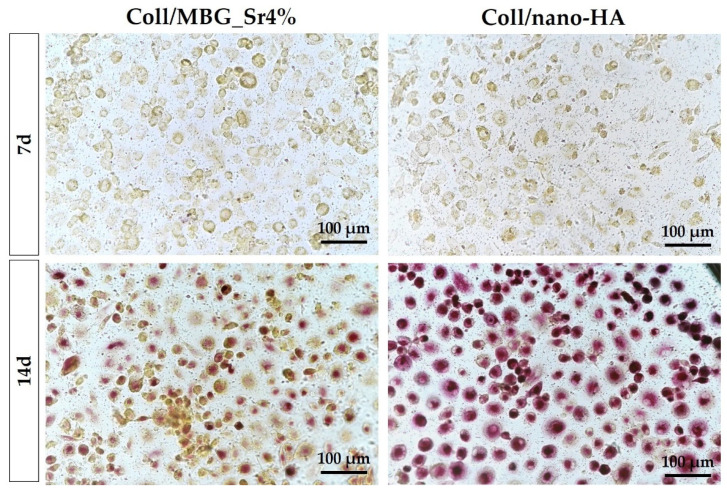
TRAP staining of PBMCs/pre-OCs on transwells in the indirect co-culture with OB-loaded materials at 7 and 14 days.

The differentiation of PBMCs toward OC precursors (pre-OCs) in the indirect co-culture with OB-loaded materials was investigated by histochemical staining of actin and multinuclearity (Figure 11), as well as TRAP (Figure 12).

The amount of pre-OCs adherent after 7 days for the two tested materials (Figure 11) confirmed the healthy state of the cells when cultured in indirect contact with the materials. Since PBMCs were seeded on a transwell PET membrane and cultured in a basal medium, their differentiation capability could have been partially affected. After 14 days, the differentiation towards pre-OCs can be appreciated: a few large cells with enhanced positivity for actin filaments can be seen, particularly for the Coll/nano-HA sample.

On day 7, a similar TRAP staining of pre-OCs on transwells is observed for both samples (Figure 12). At 14 days, a marked positivity for TRAP can be appreciated for the Coll/nano-HA sample, where pre-OCs showed stronger staining in comparison to the Coll/MBG_Sr4%-exposed cells.

## 4. Discussion

It is known that mimicking bone remodeling in vitro is a complex challenge as it involves the interplay of different cell types; nonetheless, several direct or indirect in vitro models of co-culture have been developed to investigate this synergy, also in the presence of biomaterials [13,30,31]. In this frame, the present study aimed to replicate and investigate the potential communication between OBs and OCs when in contact with biomaterials deemed promising for BTE applications [13,27]. Accordingly, the interaction of the two biomimetic collagen-based systems with human bone-derived cells was explored by introducing them in an indirect co-culture of OBs and OC precursors (PBMCs).

### 4.1. Implementation of the Co-Culture Method and Material’s Suitability

The preparation of the two biomimetic systems, Coll/nano-HA and Coll/MBG_Sr4%, started from a type I collagen-based solution to which particles of nano-HA or MBG containing Sr^2+^ ions were added to create hybrid formulations. The hydrogels were subsequently crosslinked with genipin to enhance the mechanical and thermal properties, alongside the stability, in physiological conditions. The genipin-crosslinking strategy is non-toxic for cells, as already published by the authors in previous work [20]. The materials proved to be stiff enough for cell support, showing little degradation up to 21 days, regardless of the medium used. This was also confirmed when the samples remained intact after 21 days of immersion in culture media for biological testing. The material’s biocompatibility has been previously proved by the authors and was further confirmed in this work where cells adhered and proliferated on both composites (Figure 5 and Figure 6), also giving an indication of an osteogenic potential (Figure 10).

During the development of the proposed co-culture model, the authors prioritized the distinction of the effects of the materials on the two types of cells, i.e., OBs and OCs. In this scope, it was important to maintain the two cell populations physically separated during the cell-biomaterial culture, as the indirect contact system is intended to distinguish the effects of the materials on OBs and OC precursors while allowing the natural crosstalk and paracrine signaling between these cells [13,32]. The cell viability and cell morphology were monitored, and the data were supported by studying specific markers for cell differentiation, namely ALP and TRAP activity which targeted OBs and PBMCs, respectively.

### 4.2. Cell Viability and Proliferation in Direct and Indirect Contact with the Materials

The cell viability and proliferation were monitored by means of Alamar Blue, an indirect measure of viability based on cellular metabolic activity. In particular, OBs showed a statistically significant increase from 24 h to 14 days on both materials. Cells were able to become over-confluent and to form a cell monolayer when cultured on hydrogels. Since OBs were in direct contact with the materials, these results were further supported by the Live Dead assay, which identified a negligible number of dead cells and confirmed their successful adhesion to the material’s surface.

Concerning the cell viability of PBMCs/pre-OCs, the highest value was registered at 24 h, with a decrease at 7 days followed by a statistically significant increase after 14 days. Using the Alamar Blue assay where oxidation-reduction reactions occur based on enzymatic activity, several considerations must be done [33]. In the case of PBMCs, mitochondrial enzymes are involved during the maturation of monocytes toward OCs/pre-OCs; indeed, OC differentiation from the original mononuclear progenitor cells requires high metabolic reprogramming to maintain biosynthetic substrates and energy supply [34]. In this light, the mitochondrial energy metabolism is subjected to variations and the conversion of the resorufin dye can change from 24 h to 7 days. Once the formation of pre-OCs started at 7 days, the increase of cell viability can be justified as in mature osteoclasts the mitochondria increased in size, are rich in cristae, and arranged in a complex tubular network. Potentially, a substantial increase in mitochondrial activity could affect the Alamar Blue conversion [35].

Potential cytotoxic effects of the material extracts on cells were analyzed by adding the material-conditioned medium (CM) to the cell co-culture for 24 h. The two highest concentrations of CM, i.e., 100:0 and 50:50 (CM: fresh medium), induced a decrease of cell viability, while the more diluted extracts, i.e., 25:75 and 5:95, gave the same values of the control co-culture. The present results, which are in line with the previous data published by the authors [20], confirmed that the chemical crosslinking solution and the overall sample processing do not imply any relevant cytotoxic effect, and the crosslinked samples may easily host viable cells.

### 4.3. Effect of the Biomaterials on Cell Morphology and Differentiation

The arrangement and morphology of OBs were studied by SEM. Cell-cell and cell-material contacts via pseudopodia were detected for both composite materials, and cell density appeared to increase over time, confirming the response obtained in the viability assays. For Coll/MBG_Sr4%, the cells took an elongated shape, which could derive from the high density detected by day 14, and overcrowding that is stated at day 21. By day 21, cobble-stone-shaped cells were detected, a sign of osteoblast maturation [36]. A flat shape of OBs was observed for the Coll/nano-HA, with a slight change from a spread appearance towards a fusiform shape at 21 days. The slight difference in cell morphology may be due to the different stiffness of the two substrates: Coll/MBG_Sr4% has a higher material stiffness compared to Coll/nano-HA as revealed by G′ and G″ values measured in the rheological analysis. As previously reported, strong interactions are formed between calcium ions of HA nanoparticles and carboxylate groups of collagen fibers, potentially leading to minor interactions between genipin and collagen molecules with a consequent lower degree of crosslinking [28]. It is known that the material composition and its mechanical stiffness highly influence cell adhesion. Sun et al. reported that cells become more spread out and more adhesive on substrates of higher stiffness [37] and Chatterjee et al. observed that material properties may also influence cell differentiation [38]. In our work, we found cobble-stone-shaped cells on Coll/MBG_Sr4% having the highest material stiffness, so we suggest a potentially more differentiated osteoblastic phenotype in comparison to Coll/nano-HA. In addition, the presence and shape of HA nanoparticles may affect the response of osteoblasts to the materials. As shown by Hatakeyama et al., the osteogenic differentiation of Saos-2 cells cultured on n-HAP/Col was higher than on m-HAP-Col, with different cell morphological features [39]. In the same line, when studying the morphology of the osteoblast-like cells, exposed to different kinds of HA, Shi et al. observed that the rod-like morphology, like the one used in this work, showed less favorable properties in comparison to spherical nanocrystals since the well-organized surface appears to be beneficial for filopodia protrusion [40]. Thus, the flatter morphology of OBs when in presence of HA can be related also to the shape of the nanoparticles.

The effect of the two bioactive collagen-based systems on OB differentiation was evaluated by measuring the ALP activity both in cell lysates and in culture supernatants. The evaluation of ALP activity is generally used as a marker of early differentiation of osteoblastic cells since it is expressed quite early in the osteoblast differentiation process before matrix mineralization. The data collected from the ALP assay show low values of this enzyme at 7 days, with an increase at 14 days, especially in the case of cell lysate of Coll/MBG_Sr4% which presented the highest value. The lower value of ALP activity in cell lysate registered for Coll/nano-HA, in comparison to Coll/MBG_Sr4%, may be due to the presence of nano-HA. Indeed, Ha et al. observed that nano-HA particles have a minor effect on ALP activity stimulation as cells progress along the osteoblast lineage. In our study, the osteoblasts used were derived from trabecular bone samples and consequently were already differentiated [41].

In the case of Coll/MBG_Sr4%, the higher value of ALP in the cell lysates at 7 and 14 days may be due to the presence of both Sr^2+^ and MBG. As demonstrated by Fernández et al., Sr^2+^ induces the activation of ALP due to the affinity for a specific metal-binding site, with the consequent regulation of ALP activity during the osteoid mineralization [42]. Furthermore, MBGs were found to stimulate osteogenic differentiation thanks to the release of ionic dissolution products (Si, Ca) [43,44]. Wu et al. studied the effect of MBG dissolution on the differentiation of bone marrow mesenchymal stem cells and observed that ALP levels increased in a dose-dependent manner with the various concentrations of MBG dissolution media [45]. In addition, Taherkhani et al. reported that the presence of Sr^2+^ in the MBG powder is related to osteogenic potential and increased ALP activity for osteoblast-like cells [46]

The morphology of the PBMCs was also studied over time with SEM. Given that PBMCs were cultured on the PET membrane of a transwell device, and the adhesion and differentiation activities may differ from the standard culture conditions, both the materials co-cultured indirectly with PBMCs did not affect their viability and adhesion over time. In the case of Coll/nano-HA major dimensions of pre-OCs were observed in comparison to Coll/MBG_Sr4%, together with a more pronounced macrophagic morphology and enhanced cellular spreading with ruffles. These features may be related to an advanced state of differentiation of monocytes towards OCs; however, further investigations are required to evaluate if the presence of Sr^2+^ may exert an anti-osteoclastogenic activity, as already reported in the literature. Accordingly, Baron et al. related the presence of Sr^2+^ to the reduction of the carbonic anhydrase II and vitronectin receptor expression in osteoclasts, with the consequent partial inhibition of OC differentiation [47]. Additionally, Lakkakorpi et al. observed that the presence of Sr^2+^ can alter the formation of the ruffled border during the cell attachment [48].

Considering the above, the differentiation of PBMCs towards pre-OCs was evaluated by means of actin ring formation, multinuclearity, and TRAP staining. No differences between the two materials were detected at 7 days for both actin organization and TRAP staining. Indeed, the PBMCs were mostly mononucleated and positive for actin without the formation of an evident actin ring. Instead, some positive signals were detected at 14 days, with Coll/nano-HA material showing a more intense TRAP staining in comparison to pre-OCs on Coll/MBG_Sr4% material. The reduced positivity of the OC differentiation markers in the case of Coll/MBG_Sr4% material may be due to the presence of Sr^2+^. Indeed, Sr^2+^ interferes with OC differentiation by acting on several molecular pathways, as shown by Sun et al., who found fewer TRAP-positive cells and fewer multinuclear cells in Sr^2+^-treated cells [49]. Thus, the marked TRAP-positive response of pre-OCs on Coll/nano-HA material may be related to the absence of Sr^2+^. In this light, Sr2+may exert an clastogenic effect on the cells.

In summary, Coll/MBG_Sr4% and Coll/nano-HA materials were revealed to support viability and proliferation when maintained in co-culture with human bone-derived cells, without the release of cytotoxic products, while cell maturation may be differently affected by the particles and ions introduced in the collagenous matrix.

## 5. Conclusions

It is known that the combination of collagen with bioactive inorganic phases such as mesoporous bioactive glasses and hydroxyapatite may represent versatile systems with in vitro/in vivo bioactivity able to provide multiple positive signals to bone cells and thus suitable for bone tissue engineering applications. By employing human osteoblasts and osteoclast precursors, the present study demonstrated that collagen-based systems containing Sr-enriched mesoporous bioactive glasses nanoparticles and nano-hydroxyapatite have structural and physicochemical properties able to support viability and proliferation of co-cultured human bone-derived cells, with multiple signals differently affecting the osteoblast and the osteoclast precursors. The results show that a certain level of biomimicry in cell-cell communication was achieved, proving the potential of co-culture systems as tools for the pre-screening of newly designed biomaterials.

Based on the present data and the already reported extrudability of the bioactive collagen-based formulations, a future investigation will focus on the design of 3D printed constructs able to resemble the complex architecture of bone tissue to boost the regenerative potential and the biomimicry of the developed systems for bone tissue engineering applications.

## Data Availability

Not applicable.
